# An Electronic Search Algorithm for Early Disseminated Intravascular Coagulopathy Diagnosis in the Intensive Care Unit: A Derivation and Validation Study

**DOI:** 10.7759/cureus.10972

**Published:** 2020-10-15

**Authors:** Tabinda Jawaid, Naseema Gangat, Timothy Weister, Rahul Kashyap

**Affiliations:** 1 Pathology, Mayo Clinic, Rochester, USA; 2 Hematology & Oncology, Mayo Clinic, Rochester, USA; 3 Anesthesia, Mayo Clinic, Rochester, USA; 4 Critical Care, Mayo Clinic, Rochester, USA

**Keywords:** computable phenotype, automated algorithm, disseminated intravascular coagulopathy, coagulopathy, intensive care unit

## Abstract

Aim: We aim to create and validate an electronic search algorithm for accurate detection of disseminated intravascular coagulopathy (DIC) from medical records.

Methods: Patients with DIC in Mayo Clinic’s intensive care units (ICUs) from Jan 1, 2007, to May 4, 2018, were included in the study. An algorithm was developed based on clinical notes and ICD diagnosis codes. A cohort of 50 patients was included with DIC diagnosis, its variations, and no diagnosis of DIC. Then, the next set of 50 patients was used to refine the algorithm. Results were compared with a manual reviewer and the disagreements were resolved by the third reviewer. The same process was repeated with 'revised clinical note search' for the first and second derivation cohort with additional exclusion terms. The obtained sensitivity and specificity were reported. The generated algorithm was applied to another set of 50 patients for validation.

Results: In the first derivation cohort- DIC search by clinical notes and diagnosis codes had 92% sensitivity and 100% specificity. Sensitivity dropped to 71% in the second cohort although specificity remains the same. Therefore, the algorithm was refined to clinical notes search only. The revised search was reapplied to first and second derivation cohorts and results obtained for the first derivation were the same but 91.3% sensitive and 100% specific for the second derivation. The search was locked and applied in the validation cohort with 95.8% sensitivity and 100% specificity, respectively.

Conclusion: The revised clinical note based electronic search algorithm was found to be highly sensitive and specific for DIC during the corresponding ICU duration.

## Introduction

Disseminated intravascular coagulopathy (DIC) can be classified into two parts: thrombotic; formation of multiple small blood clots (thrombi) in the different vessels of the body leading to reduced blood flow to organs, hence, damage of multiple body systems, and hemorrhagic, with fewer platelets and clotting factor, the body becomes prone to severe hemorrhage (internal and external). Sometimes DIC can remain clinically silent and undetected in laboratory findings but can only be identified by coagulation profile (elevated prothrombin time [PT], activated partial thromboplastin time [aPTT]) [1]. Clinically, DIC can present with dyspnea, acrocyanosis, pallor, severe muscle, back, and abdominal pain, oliguria, multi-organ convulsion, and coma. Clinical presentation varies among patients. Other laboratory workups that support DIC diagnosis are complete blood count (anemia, thrombocytopenia), elevated fibrinogen degraded product, d-dimer, and reduced fibrinogen level, anti-thrombin III, and protein C.

The common causes for the development of disseminated intravascular coagulopathy are sepsis or severe infection, malignancy, trauma, and obstetric disorders such as placental abruption, amniotic fluid embolism, etc. [2-4]. Severe sepsis is the common cause of DIC due to hyperactivation of the complement system and more than one-third of sepsis patients are reported to develop DIC [5-6]. According to the Japanese Association for Acute Medicine (JAAM) and the International Society of Thrombosis and Hemostasis (ISTH), DIC patients have higher mortality among patients admitted in the hospital [7].

An electronic medical record is a tool that has been implemented and used for more than 30 years to modernize clinical medicine and to reduce cost and time in health care researches [8]. However, an effective method is required to identify, capture, and extract particular data in a short period. Algorithms have been derived and validated previously such as identification of post-operative complications, cognitive impairment, sepsis, continuous renal replacement therapy, mechanical ventilation initiation, emergent intubation, Charlson comorbidities, and extubation failure, but no strategy has been developed for DIC diagnosis identification [9-16].

We aim to develop an automated algorithm for retrospective research studies that will require DIC diagnosis identification. The algorithm will provide accurate detection of diagnosis in a shorter period compared to manual chart review.

## Materials and methods

This study was approved by the Institutional Review Board of Mayo Clinic to use patients' medical records from the database. Only those patients with Minnesota Research Authorization were included in the review.

Study population

**Figure 1:**
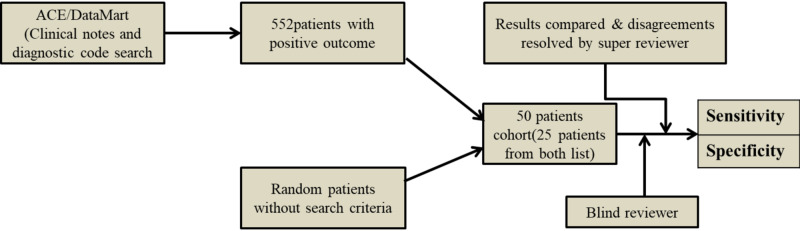
Flow chart of study derivation and validation cohorts 1^st^ and 2^nd^ derivation cohort with clinical notes and diagnostic code search ACE: Advanced Cohort Explorer

The study consisted of 18 years or above and was admitted to Mayo Clinic’s intensive care unit (ICU) from Jan 1, 2007, to May 4, 2018. One hundred fifty out of 552 patients were sampled that met DIC's search criteria from clinical notes and diagnostic code search. Out of three cohorts, each cohort consisted of 50 patients (25 patients that met the search criteria and the remaining 25 patients without search criteria). The first cohort was used to derive an electronic search algorithm followed by the second cohort derivation to refine an algorithm (Figure 1).

**Figure 2:**
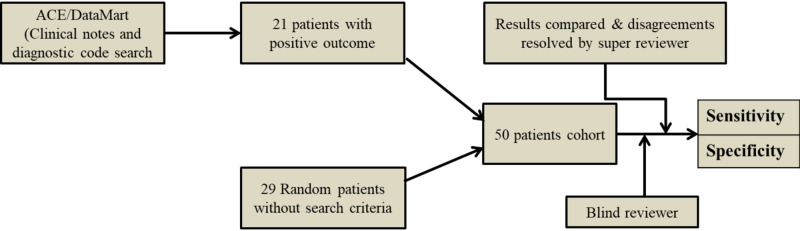
Flow chart of study derivation and validation cohorts 1^st^ derivation cohort with revised clinical notes search ACE: Advanced Cohort Explorer

**Figure 3:**
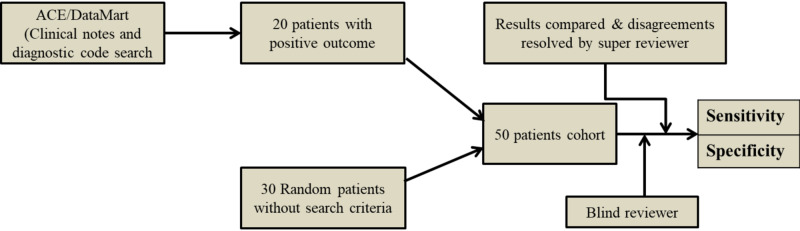
Flow chart of study derivation and validation cohorts 2^nd^ derivation cohort with revised clinical notes search ACE: Advanced Cohort Explorer

**Figure 4:**
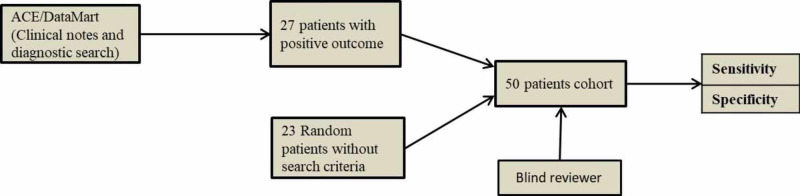
Flow chart of study derivation and validation cohorts Validation cohort with revised clinical note search algorithm ACE: Advanced Cohort Explorer

The same method was repeated with the ‘revised clinical notes’ search only, and additional exclude terms due to inconsistency in results obtained from the clinical notes and diagnostic code search. The first derivative cohort had 21 patients who met the search criteria and 19 patients without search criteria (Figure 2). The second derivative had cohort 20 patients that met the search criteria and the remaining 20 patients without search criteria (Figure 3). The validation cohort had 27 patients that met the search criteria and the remaining 13 patients without search criteria (Figure 4). This revision consisted of algorithm refinement with additional exclude terms applied with the search algorithm.

Automated electronic search strategy

Data for the retrospective study was used from the Mayo Clinic ICU DataMart and Data Platform, an extensive data warehouse containing a near real-time normalized replica of Mayo Clinic’s electronic medical records (EMR). These databases contain patient information and their laboratory test results, clinical and pathological information from sources within the institution, and have been previously validated. A web-based software toolset, Advanced Cohort Explorer (ACE), was used for data access. 

The search terms as ‘disseminated intravascular coagulation,’ ‘disseminated intravascular coagulopathy,’ ‘DIC,’ ‘defibrination syndrome,’ ‘intravascular coagulopathy,’ ‘consumption coagulopathy,’ ‘consumptive coagulopathy,’ or ‘fibrinolysis’ were abstracted and terms like ‘no’ ‘negative,’ ‘history,’ ‘ruled out’ were removed. Cases were identified as having or not having DIC. The algorithm was refined continuously in several iterations with additional exclusion terms as follows: 1. Fibrinolysis and liver disease, 2. Fibrinolysis and cirrhosis, 3. DIC and panel, 4. DIC and profile, 5. DIC and HIT.

This improved the sensitivity and specificity of the derivation subset to more than 90%. The phenotype algorithm was validated using sensitivity and specificity calculated by comparing the results to the gold standard obtained by manual review (Figure 1-4). The search algorithm for the cohort was done under the supervision of an independent critical care researcher.

Manual data abstraction for gold standard

Cohorts established were reviewed by a blind reviewer. The manual review consisted of clinical features and laboratory values- D-dimer, fibrinogen level, PT, APTT, platelet count, and hemoglobin, peripheral smear, fibrin monomer, lactate dehydrogenase (LDH), indirect bilirubin, and haptoglobin during the ICU stay [17].

A blind reviewer manually reviewed the two derivative cohorts established from clinical notes and diagnostic searches through ACE and DataMart. A third reviewer resolved the conflicts with access to electronic data, manual review results, and medical charts [16]. Once conflicts were resolved, sensitivity and specificity were derived (Figure 1).

A blind reviewer reviewed two derivatives cohorts established from ’revised clinical notes’ search only criteria, and the third reviewer resolved conflicts. Diagnostic code was not included in a second search because it includes patients with coagulopathy but likely without DIC. After reviewing, sensitivity/specificity was derived. A validation cohort of 50 patients derived from ACE was validated by a blind reviewer and compared with a gold standard for the study to validate the sensitivity and specificity of the algorithm generated and refined (Figure 1).

Statistical analysis

The computable phenotype's sensitivity and specificity were calculated by comparing the results to the gold standard obtained by manual review of the charts. We used JMP statistical software 14.0 Pro (SAS Corp., Cary, NC, USA).

## Results

In the first derivation cohort, the algorithm achieved a sensitivity of 92% and 100% specificity by clinical notes and diagnosis codes. There were disagreements between the manual review and algorithm extracted dataset, but it was resolved by the third reviewer who had access to EMR and medical records. The supervised algorithm was reapplied to the second cohort derivative, and the results achieved had a sensitivity of 71% sensitivity and 100% specificity with clinical notes and diagnosis codes search. Due to a drop in sensitivity, data was derived again with ‘clinical notes’ search only, and data was revised. In the first cohort, sensitivity/specificity remains unchanged compared to clinical notes and diagnosis code search. Although, in second cohort derivatives, sensitivity and specificity increased to 93.8% and 100% (Table 1). This algorithm was refined and generated.

**Table 1:** Automated algorithm generated sensitivity and specificity for disseminated intravascular coagulopathy (DIC)

	1^st^ Derivation		2^nd^ Derivation		Validation	
	Sensitivity	Specificity	Sensitivity	Specificity	Sensitivity	Specificity
Clinical Notes and Diagnosis Codes	92%	100%	71%	100%	-	-
Diagnostic Codes only	85.2%	91.3%	74.2%	73.7%	-	-
Revised Clinical Notes	93.1%	100%	93.8%	100%	95.8%	100%

The validation of an independent cohort of 50 patients obtained through the generated algorithm was completed by data extracted by the electronic database and manual reviewer. The results obtained had a sensitivity and specificity of 95.8% and 100% (Table 1).

## Discussion

The purpose of the study was to introduce a faster and reliable method of retrospective data extraction of DIC patients from electronic medical records. The data obtained from the automated algorithm was validated by comparing it with the blinded manual reviewer and revising the reviewed data with clinical notes. As anticipated, the results demonstrated the automated algorithm was more effective when compared with the manual review of the data.

This method has been previously used for septic patients and it may help researchers to save time and cost from a manual chart review process [12]. This effective method of automated algorithm generation provides an opportunity for other institutions to replicate algorithms for DIC and other similar entities.

The strength of the study is that the method of electronic data extraction by the automated algorithm is cost-effective, faster, and more reliable compared to the manual reviewer. The total time frame for data extraction (diagnostic codes and clinical notes) was 15 hours estimated and 40 hours for data review of each set of 50 patients. Hence, it is assumed that data extraction for DIC patients will consume less time in the future.

There are several limitations to the study as well. The patient data was from single-center ICU patients only. The data was collected retrospectively. With a possibility of some patients dying before performing tests to diagnose DIC and the real-time DIC diagnosis determination may have missed out. It was also noticed in the 2nd derivation cohort that with diagnosis codes search, sensitivity, and specificity were reduced when compared to clinical notes and diagnosis codes search sensitivity-specificity (Table 1). This indicates the unreliability of the use of diagnosis codes. Also, a database like ICU DataMart and applications like Advanced Cohort Explorer systems used for data extraction is restricted to Mayo Clinic, but this validated approach can be applied to any institutional database and using an appropriate application to make it generalizable.

## Conclusions

In conclusion, the automated algorithm has higher sensitivity and specificity in establishing a diagnosis of DIC in medical charts among ICU patients and it can be used for future researches based on DIC in ICU patients to accurately detect the population of interest and could save time to manually review data.
